# Biopsy-Proven Prostatic Metastasis From Diffuse-Type Gastric Adenocarcinoma Presenting With Bilateral Malignant Ureteric Obstruction: A Case Report

**DOI:** 10.7759/cureus.115602

**Published:** 2026-09-01

**Authors:** Sami Atiq, Ahmed Anber, Praveen Subbiah, Wasim Mahmalji, Eesha Tehreem

**Affiliations:** 1 Urology, Hereford County Hospital, Hereford, GBR; 2 Urology, Oxford University Hospitals NHS Foundation Trust, Oxford, GBR; 3 Urology, Barking Havering and Redbridge NHS Trust, London, GBR; 4 Urology, Cambridge University Hospitals NHS Trust, Cambridge, GBR; 5 Family Medicine, Countess of Chester Hospital, Chester, GBR

**Keywords:** bilateral hydronephrosis, diffuse-type gastric cancer, gastric adenocarcinoma, malignant ureteric obstruction, prostatic metastasis

## Abstract

Metastatic involvement of the prostate and bladder trigone from gastric adenocarcinoma is exceedingly rare. We report the case of a 38-year-old man with previously treated diffuse-type gastric adenocarcinoma who developed bilateral hydroureteronephrosis during surveillance. Cystoscopy demonstrated an Inflamed prostatic urethra with early papillary changes and abnormal trigonal mucosa with bilaterally narrowed ureteric orifices. Retrograde studies revealed bilateral mid-ureteric strictures without intraluminal lesions. Biopsies from the prostatic urethra and trigone confirmed metastatic gastric adenocarcinoma. Ureteric stents failed to provide adequate drainage, necessitating bilateral nephrostomy insertion. This case highlights an unusual pattern of lower genitourinary metastasis from diffuse gastric cancer and underscores the importance of histological confirmation and multidisciplinary management.

## Introduction

Gastric adenocarcinoma remains a major cause of cancer-related mortality worldwide [1]. Diffuse-type gastric adenocarcinoma is characterised by infiltrative growth and a distinct pattern of metastatic dissemination [2]. Common metastatic sites include the liver, peritoneum, lungs, and bones [3].

Secondary solid tumours involving the prostate are uncommon. In a clinicopathological series of 51 cases, secondary solid neoplasms accounted for 2.1% of prostatic neoplasms identified in surgical specimens [4]. Metastatic gastric adenocarcinoma involving the prostate has been reported only rarely, predominantly in isolated case reports [5]. Involvement of the prostatic urethra and bladder trigone is particularly unusual.

We present a rare case of biopsy-confirmed metastatic diffuse-type gastric adenocarcinoma involving the prostatic urethra and bladder trigone and presenting with bilateral malignant ureteric obstruction.

## Case presentation

A 38-year-old man with a history of diffuse-type gastric adenocarcinoma was under oncological surveillance following previous treatment. The tumour was human epidermal growth factor receptor 2 (HER2)-negative, had a programmed death-ligand 1 combined positive score of 0, and demonstrated proficient mismatch repair status. In 2022, he received perioperative fluorouracil, leucovorin, oxaliplatin, and docetaxel (FLOT) chemotherapy, followed by robotic total gastrectomy. Histopathological examination of the resection specimen demonstrated ypT4aN2 disease, with metastatic involvement of 3 of 38 lymph nodes.

In May 2025, he presented with a several-month history of bilateral loin discomfort and lower urinary tract symptoms. Urine microscopy and culture showed no evidence of infection. In view of his new lower urinary tract symptoms and history of metastatic malignancy, serum prostate-specific antigen (PSA) was measured as part of the assessment for possible primary or secondary prostatic involvement. The PSA was 1.6 ng/mL, within the laboratory reference range. Renal function was preserved, with an estimated glomerular filtration rate of 82 mL/min/1.73 m². Given the bilateral loin discomfort and clinical concern for upper urinary tract obstruction or ureteric calculi, a non-contrast CT KUB (kidneys, ureters, and bladder) was selected as the initial investigation. This demonstrated bilateral hydronephrosis and unexpected pelvic soft-tissue abnormalities, prompting contrast-enhanced CT urography. CT urography confirmed bilateral hydroureteronephrosis and raised the possibility of a filling defect within the right renal pelvis (Figure 1A).

**Figure 1:**
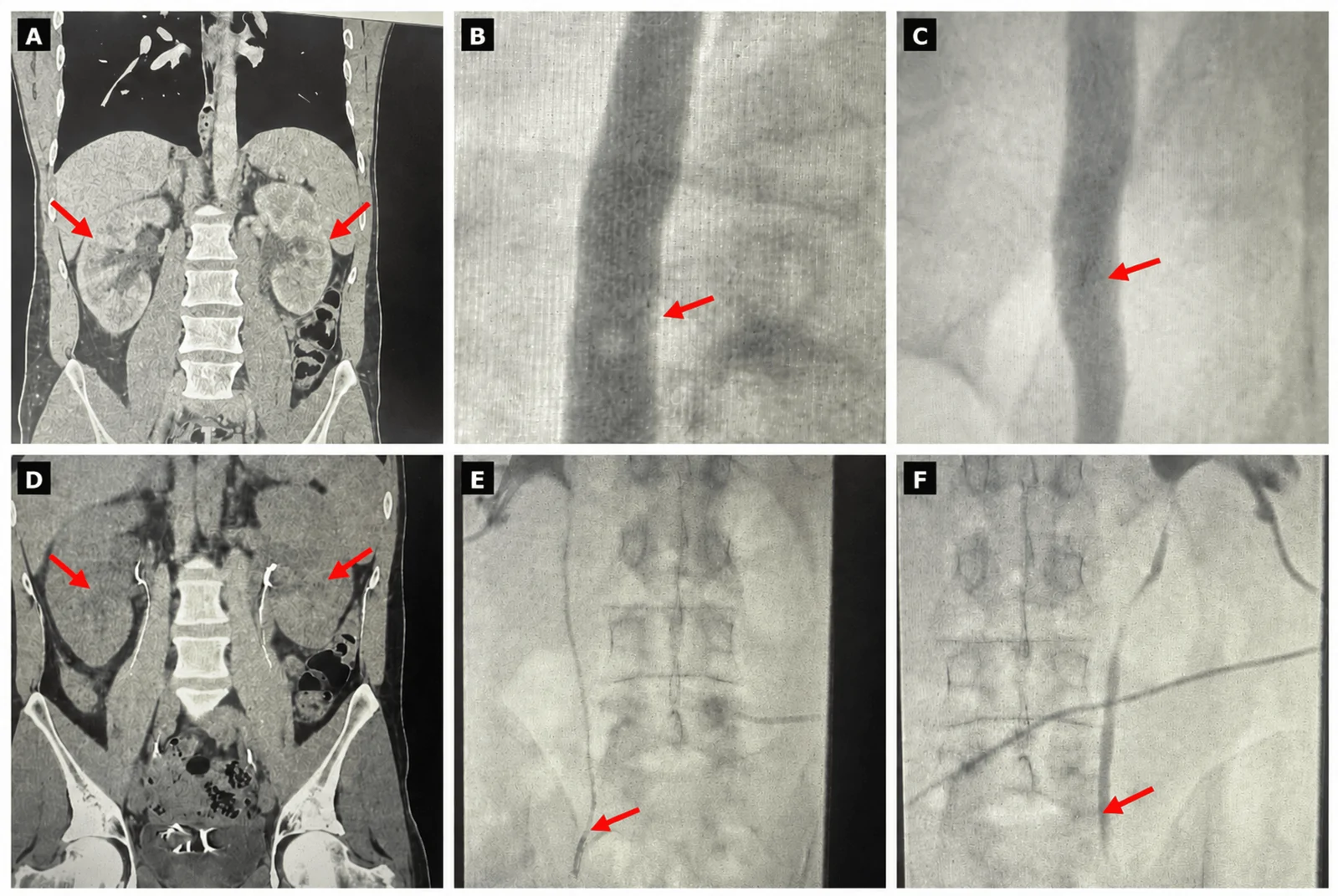
Radiological progression of bilateral malignant ureteric obstruction secondary to metastatic diffuse-type gastric adenocarcinoma (A) Coronal CT urogram demonstrating bilateral hydroureteronephrosis; arrows highlight the dilated bilateral renal collecting systems. (B) Left retrograde ureteropyelogram demonstrating hydroureteronephrosis; the arrow highlights the hydroureter. (C) Right retrograde ureteropyelogram demonstrating hydroureteronephrosis; the arrow highlights the hydroureter. (D) Coronal CT KUB (kidneys, ureters, and bladder) following bilateral ureteric stent insertion demonstrating persistent bilateral hydronephrosis despite satisfactory stent position; arrows highlight the persistent dilatation of the collecting systems. (E) Left antegrade nephrostogram demonstrating passage of contrast into the urinary bladder through a narrow and mildly irregular ureter; the arrow identifies the severe distal ureteric narrowing. (F) Right antegrade nephrostogram demonstrating passage of contrast into the urinary bladder, with marked ureteric narrowing below the level of the sacroiliac joint; the arrow identifies the severely narrowed distal segment.

The case was discussed at the urology multidisciplinary team meeting, and the patient subsequently underwent examination under anaesthesia, cystoscopy, bilateral retrograde ureteropyelography, ureteroscopy, biopsy, and bilateral ureteric stent insertion. Examination under anaesthesia demonstrated a small prostate without a palpable or fixed pelvic mass. The anterior urethra was unremarkable; however, cystoscopy revealed inflamed and vascular mucosa within the prostatic urethra, with early papillary changes. The bladder trigone appeared abnormal, and both ureteric orifices were markedly tight. The bladder was of large capacity but showed no evidence of trabeculation.

Bilateral retrograde ureteropyelography demonstrated severe bilateral hydroureteronephrosis with focal mid-ureteric narrowing; no contrast extravasation was observed. Ureteroscopy identified tight, ischaemic-appearing segments within both mid-ureters that were difficult to traverse. No intraluminal tumour was visualised within either ureter or renal pelvis, including the right renal pelvis where a possible filling defect had been suggested on CT urography. Biopsies were obtained from the abnormal areas of the prostatic urethra and bladder trigone.

Histopathological examination of the bladder trigonal and prostatic urethral biopsies demonstrated a poorly differentiated invasive carcinoma involving the subepithelial stroma (Figures 2A, 2B). The tumour cells were arranged in sheets and single-cell files. A small number of cells showed cytoplasmic vacuolation, with focal extracellular mucin. The overlying urothelium demonstrated features of cystitis glandularis without evidence of carcinoma in situ. Immunohistochemical analysis demonstrated diffuse, strong nuclear expression of CDX2 and patchy CK7 positivity (Figures 3A, 3B). The tumour cells were negative for GATA3, CK20, prostate-specific antigen, thyroid transcription factor 1, oestrogen receptor, and leukocyte common antigen. This immunophenotypic profile favoured an alimentary tract primary and argued against primary urothelial or prostatic carcinoma. The reporting pathologist reviewed the patient’s previous gastric biopsy, which had been diagnosed as diffuse-type gastric adenocarcinoma, and identified morphological resemblance between the gastric and urinary tract tumours. In the context of the patient’s known gastric malignancy, these findings were interpreted as metastatic diffuse-type gastric adenocarcinoma involving the prostatic urethra and bladder trigone. The interpretation was independently reviewed by a second pathologist, who concurred.

**Figure 2:**
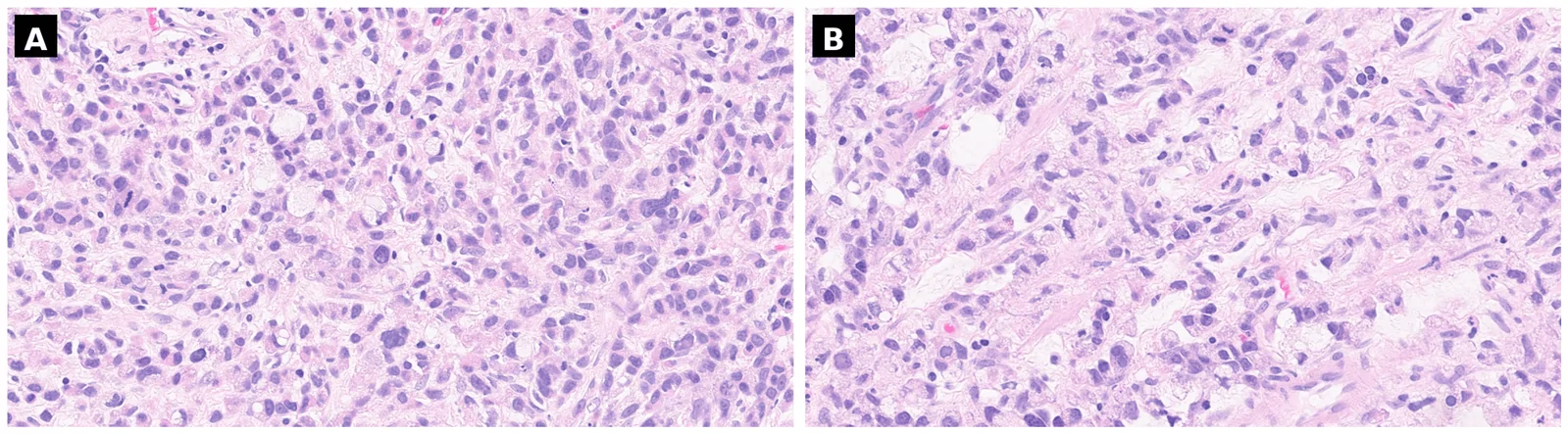
Histopathological appearance of the bladder trigonal and prostatic urethral biopsies (A) High-magnification haematoxylin and eosin-stained section from the bladder trigonal biopsy, demonstrating poorly differentiated invasive carcinoma involving the subepithelial stroma. (B) High-magnification haematoxylin and eosin-stained section from the prostatic urethral biopsy showing poorly differentiated invasive carcinoma, with tumour cells arranged in sheets and single-cell files, focal cytoplasmic vacuolation, and areas suggestive of extracellular mucin.

**Figure 3:**
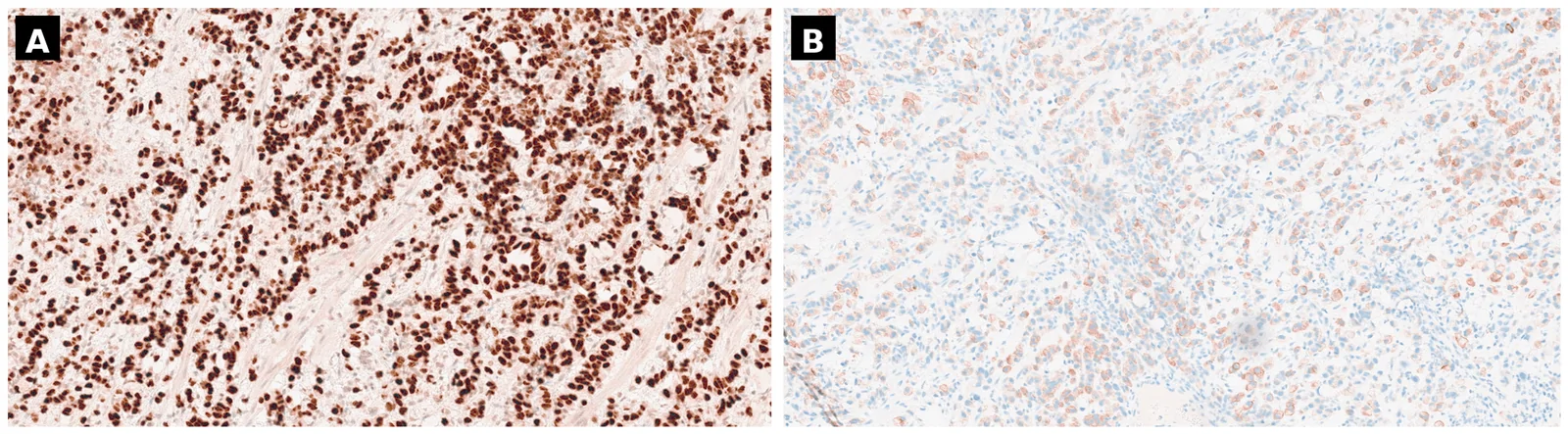
Immunohistochemical profile of the metastatic tumour (A) Diffuse strong nuclear CDX2 expression within the tumour cells, supporting gastrointestinal differentiation. (B) Patchy CK7 positivity within the tumour cells.

Although the ureteroscope could not be advanced across the tight mid-ureteric segments, a Sensor guidewire was successfully negotiated beyond each narrowed segment into the renal pelvis under fluoroscopic guidance. Bilateral 6-Fr multi-length ureteric stents were subsequently deployed over the guidewires. Retrograde internal drainage was attempted initially because guidewire access had been achieved, the patient was clinically stable, and internal stenting avoided immediate external drainage. A follow-up CT KUB performed approximately six weeks later demonstrated persistent bilateral hydronephrosis despite satisfactory stent position (Figure 1D), indicating inadequate internal drainage. Bilateral percutaneous nephrostomies were therefore inserted, and the ureteric stents were removed. Following confirmation of metastatic recurrence in May 2025, the patient was reviewed by the oncology team and commenced on palliative capecitabine and oxaliplatin (CAPOX) chemotherapy. He completed six cycles of treatment. Overall, treatment was tolerated reasonably well, although the final cycle was associated with increased fatigue and peripheral neuropathy, which subsequently began to improve. A response-assessment CT performed on 14 January 2026 demonstrated improvement in the previously noted thickening and enhancement of the left upper ureter, with stable appearances around the mesorectal fascia and no new radiological abnormalities. The oncology team considered this to represent a good response to treatment and planned repeat surveillance imaging after three months. At the oncology review in January 2026, the patient reported significant discomfort related to the bilateral nephrostomies, including pain and pruritus around both nephrostomy sites, despite review by the clinical nurse specialist team and trials of alternative dressings and supportive measures. He had also experienced a recent urinary infection associated with one of the nephrostomies and reported dysuria. Urine microscopy, culture and sensitivity identified Escherichia coli susceptible to nitrofurantoin, and a further course of nitrofurantoin was prescribed. In March 2026, bilateral nephrostography demonstrated antegrade passage of contrast through both ureters into the urinary bladder. However, the left ureter was narrow and mildly irregular throughout, while the right upper ureter was slightly wider but became markedly narrowed below the level of the sacroiliac joint. Both distal ureters were therefore of extremely narrow calibre. Contrast injection required pressure and was associated with flank pain, and the reporting radiologist questioned whether effective internal drainage would be achievable. Despite these concerning findings, both nephrostomies were clamped as a trial, and the patient was advised to seek urgent medical attention if symptoms of infection or obstruction developed. The patient subsequently developed recurrent obstructive and infective symptoms, unclamped the nephrostomies at home, and returned to hospital, where recurrent obstruction complicated by pyelonephritis was diagnosed. Free nephrostomy drainage was resumed, and a decision was made to maintain long-term bilateral nephrostomies with scheduled exchanges. At the most recent follow-up, the nephrostomies remained in situ, and the patient continued under oncological follow-up with ongoing nephrostomy care.

## Discussion

Secondary solid tumours involving the prostate are uncommon and account for only a small proportion of prostatic malignancies [4]. Metastatic gastric adenocarcinoma involving the prostate has been documented in isolated case reports [5]. The present case is particularly unusual because diffuse-type gastric adenocarcinoma involved the prostatic urethra and bladder trigone and presented with bilateral malignant ureteric obstruction.

Diffuse-type gastric adenocarcinoma has an infiltrative growth pattern and a metastatic distribution distinct from that of intestinal-type gastric carcinoma [2,6]. In the present case, mesorectal fascial thickening, pelvic soft-tissue stranding, bilateral mid-ureteric strictures, and the absence of a discrete intraluminal tumour were consistent with an infiltrative metastatic process. However, the precise route by which the tumour reached the prostatic urethra and bladder trigone could not be established.

Establishing the diagnosis required histological and immunohistochemical confirmation because the presenting symptoms and radiological findings were nonspecific. Lower urinary tract symptoms and obstructive uropathy in a patient with a history of gastric malignancy may initially be attributed to benign prostatic disease, primary urothelial malignancy, treatment-related fibrosis, or recurrent metastatic disease. In this patient, the normal prostate-specific antigen level and small prostate made primary prostatic adenocarcinoma less likely but did not establish the origin of the abnormal tissue.

Cystoscopy demonstrated abnormal vascular mucosa within the prostatic urethra and an abnormal bladder trigone, allowing targeted biopsies to be obtained. Histopathological examination demonstrated a poorly differentiated invasive carcinoma within the subepithelial stroma. Diffuse CDX2 expression and patchy CK7 positivity supported gastrointestinal differentiation, while the absence of GATA3 and prostate-specific antigen expression argued against primary urothelial and prostatic carcinoma, respectively. The reporting pathologist identified morphological resemblance between the urinary tract tumours and the patient’s previous gastric biopsy. In conjunction with the immunophenotypic profile and known clinical history, these findings supported metastatic recurrence of diffuse-type gastric adenocarcinoma. The reporting pathologist identified morphological resemblance between the urinary tract tumours and the patient’s previous gastric biopsy. In conjunction with the immunophenotypic profile and known clinical history, these findings supported metastatic recurrence of diffuse-type gastric adenocarcinoma.

The discrepancy between the suspected right renal pelvic filling defect on CT urography and the absence of an intraluminal tumour at ureteroscopy demonstrates the importance of direct endoscopic assessment when imaging findings are inconclusive. Infiltrative or extrinsic malignant disease may cause obstruction without producing a visible intraluminal lesion.

Malignant ureteric obstruction is associated with advanced malignancy and carries important prognostic implications [7]. Management should be individualised according to symptoms, renal function, performance status, anticipated oncological treatment, overall disease burden, and patient preference. Both retrograde ureteric stenting and percutaneous nephrostomy are recognised methods of urinary decompression in patients with malignant ureteric obstruction [8]. In this case, bilateral ureteric stents did not provide adequate drainage, necessitating bilateral percutaneous nephrostomy placement. A subsequent clamping trial resulted in recurrent obstruction and pyelonephritis. This case also demonstrates that radiographic passage of contrast into the bladder during nephrostography does not necessarily confirm adequate physiological ureteric drainage. In this patient, contrast traversed both ureters only through severely narrowed segments, required pressurised injection, and provoked flank pain; these findings indicated that sustained internal drainage was unlikely to be effective.

In retrospect, given the severe bilateral malignant ureteric narrowing and the radiologist’s concern regarding the feasibility of internal drainage, nephrostomy clamping should not have been undertaken. Following the unsuccessful trial, long-term free nephrostomy drainage was maintained.

## Conclusions

This case demonstrates a rare presentation of diffuse-type gastric adenocarcinoma involving the prostatic urethra and bladder trigone and causing bilateral malignant ureteric obstruction. In selected patients with a history of gastric cancer and otherwise unexplained obstructive uropathy, lower genitourinary metastatic involvement may form part of the broader differential diagnosis, particularly when no intraluminal upper urinary tract lesion is identified. Histopathological confirmation and multidisciplinary coordination are important for establishing the diagnosis and planning urinary drainage and oncological management.

## References

[1] Sung H, Ferlay J, Siegel RL, Laversanne M, Soerjomataram I, Jemal A, Bray F (2021). Global cancer statistics 2020: GLOBOCAN estimates of incidence and mortality worldwide for 36 cancers in 185 countries. CA Cancer J Clin.

[2] LA P (1965). The two histological main types of gastric carcinoma: diffuse and so-called intestinal-type carcinoma. An attempt at a histo-clinical classification. Acta Pathol Microbiol Scand.

[3] Riihimäki M, Hemminki A, Sundquist K, Sundquist J, Hemminki K (2016). Metastatic spread in patients with gastric cancer. Oncotarget.

[4] Bates AW, Baithun SI (2002). Secondary solid neoplasms of the prostate: a clinico-pathological series of 51 cases. Virchows Arch.

[5] Ni YJ, Mei GT, Wang CT, Tung YP (2013). Gastric adenocarcinoma with prostate metastasis. Urol Sci.

[6] Koemans WJ, Luijten JC, van der Kaaij RT, Grootscholten C, Snaebjornsson P, Verhoeven RH, van Sandick JW (2020). The metastatic pattern of intestinal and diffuse type gastric carcinoma - a Dutch national cohort study. Cancer Epidemiol.

[7] Lienert A, Ing A, Mark S (2009). Prognostic factors in malignant ureteric obstruction. BJU Int.

[8] Ku JH, Lee SW, Jeon HG, Kim HH, Oh SJ (2004). Percutaneous nephrostomy versus indwelling ureteral stents in the management of extrinsic ureteral obstruction in advanced malignancies: are there differences?. Urology.

